# Special Survival Strategy of First-Instar Scorpions Revealed by Synchronous Molting Behavior from Social Facilitation of Maternal Care and Reciprocal Aggregation

**DOI:** 10.3390/insects15090726

**Published:** 2024-09-21

**Authors:** Yiyuan Guo, Songryong Li, Sijia Lu, Xinrong Wang, Zhijian Cao, Yingliang Wu

**Affiliations:** College of Life Sciences, Wuhan University, Wuhan 430072, China; 2019202040010@whu.edu.cn (Y.G.); lisongryong2020@163.com (S.L.); 2022202040009@whu.edu.cn (S.L.); 2022202040010@whu.edu.cn (X.W.); zjcao@whu.edu.cn (Z.C.)

**Keywords:** synchronous molting, aggregate molting, maternal care, reciprocal aggregation, *Mesobuthus martensii*, survival rate, first-instar scorpion

## Abstract

**Simple Summary:**

The ecdysis of arthropods is an essential process for survival, growth, and reproduction. Independent molting behavior is widely adopted by most arthropods, but synchronous molting behavior is uncommon in some groups of arthropods. Synchronous molting behavior is sometimes caused by stimuli from the physical environment and food supply and sometimes involves social facilitation. As some of the earliest terrestrial arthropods, newborn scorpions exhibit unique synchronous molting behavior induced by social facilitation based on maternal care and reciprocal aggregation. Before molting, strong maternal care provided by mother scorpions helps newborn scorpions climb onto her back, which results in a survival rate of approximately 100% after molting. Moreover, aggregate molting behavior greatly increases the survival rate of newborn scorpions via collaborative molting by enabling the interconnection of pedipalps, legs, or tails among the scorpions, whether they are on the back of the mother scorpion or not. Our results highlight the novel role of social facilitation based on maternal care and reciprocal aggregation during the simultaneous molting of arthropods, which markedly enhances our knowledge of the extraordinary survival strategy of ancient arthropods during the molting process.

**Abstract:**

Ecdysis is a well-known developmental feature among arthropods. Because the aggregate and synchronous molting of first-instar scorpions is markedly different from the common independent molting behavior of older scorpions and most arthropods, knowledge on the biological benefits of the unusual behavior of first-instar scorpions remain limited. Before the molting of newborn scorpions, their mothers exhibited a remarkable ability to efficiently locate the fallen offspring and help them climb onto their back, which was supported by strong maternal behavior because they climbed more swiftly than the 7-day postpartum scorpions. Most newborn scorpions molted and survived on the mother’s back, with a survival rate of approximately 100%, and most newborn scorpions survived via aggregate molting behavior on sand in the absence of mothers (89.83% ± 1.91%). The important role of the mother scorpion was further highlighted in mothers with one to five first-instar scorpions. While all first-instar scorpions individually or reciprocally molted and survived on the mother’s back, only 52.00% ± 7.14% to 79.20% ± 4.24% of newborn scorpions isolated from the mother could individually or reciprocally molt and survive on the sand, and the aggregated states of first-instar scorpions strengthened as their numbers on sand increased before molting. These results highlight collaborative molting as an evolutionary driving force for newborn scorpions. Taken together, both maternal care and collaborative aggregate molting behavior enhanced the survival of first-instar scorpions before and after molting, and these benefits for first-instar scorpions play essential and evolutionary roles in scorpion survival.

## 1. Introduction

Arthropods, including insects, myriapods, chelicerates, and crustaceans, have a chitinous exoskeleton that plays an important role in their evolutionary success by providing protection and minimizing desiccation [1,2]. Nevertheless, these hardened exoskeletons must be molted and resynthesized periodically because they limit the expansion of body size during growth and development [3]. As an essential process, ecdysis must occur throughout life to maintain survival, growth, and reproduction in arthropods. However, molting is a risky behavior because the new exoskeleton is too soft for protection against predators and conspecifics. Therefore, arthropods have developed different behaviors to overcome this problem. Independent molting behavior is widely adopted by most arthropods, but synchronous molting behavior is also observed in modern social and sub-social groups of arthropods, such as spiders [4], shrimps [5], prawns [6], crabs, and springtails [7,8], whose presence is being increasingly observed in fossil records (e.g., trilobites [9,10,11,12,13], eurypterids [14,15], megacheirans [16], and other crustaceomorpha [9,16]).

Scorpions are among the earliest terrestrial arthropods. Molting events have been described for extinct sea scorpions, which are the presumed ancestors of extant scorpions [17]. In the Silurian, aquatic scorpions became amphibious or terrestrial due to the presence of scorpion-like leg basitarsus [18,19]. Scorpions basically retain their external morphology and molting behavior and are referred to as the evincible fossil of the terrestrial scorpion, which is smaller in size [19]. In contrast to most annual arthropods, scorpions are exceptional animals in terms of potential longevity and the duration of the juvenile stage [20,21] because they are among the longest-lived arthropods and may live up to 25 years [20]. As rare examples of viviparity in arachnids, first-instar scorpions climb onto the mother’s back after birth to continue development and molt for the first time [20,21]. The young second-instar scorpions then disperse to assume an independent existence. During most scorpion molting events, the growth of exoskeleton structures occurs with little change in morphology [22], but the main differentiation of scorpion-like exuvial morphology can be observed during molting between first- and elder-instar scorpions. Afterward, they usually continue to molt several times before reaching maturity during an extraordinarily long period that lasts 6 to 83 months [20,21]. Maternal care usually occurs from birth to the first molting, i.e., the first-instar scorpion [23,24]. It is quite obvious that in the life history of first-instar scorpions, the effects of maternal care and the first molting on survival and growth are particularly important. This work used the widely distributed *Mesobuthus martensii* scorpions in China to investigate the potential events associated with special maternal care, as well as the unusual synchronous molting behavior of first-instar scorpions. The benefits of maternal care and reciprocal aggregation were revealed and highlight the critical role of these processes in scorpion survival.

## 2. Materials and Methods

### 2.1. Animals and Maintenance

Pregnant *Mesobuthus martensii* scorpions were obtained from the scorpion breeding base of Shiyan city in Hubei Province, China, and the scorpions were raised in plastic boxes with a supply of water and food (yellow mealworms) in summer. During parturition, every pregnant female scorpion individually occupied a plastic cup (5.50 cm in base diameter, 12.00 cm in height, and 8.50 cm in top diameter) with approximately 3.00 cm of moist sand covering the bottom of the cup, where the newborn scorpions could avoid being injured by other scorpions. The delivery time of pregnant females was determined via observation every two hours to guarantee the simultaneous growth and development of first-instar scorpions in every group. After parturition, mothers spend seven days accompanying their offspring, and the first instars took approximately three days to transfer to the second instars [25]. For the behavioral experiments with newborn scorpions in the absence of their mother, the newborn scorpions were selected on the mother’s back within twelve hours after parturition, and a soft and fine brush was used to gently separate the first-instar scorpions from the mother’s back and transfer them to a new moist-sand-covered cup with 7-day postpartum or dead scorpions to track their behavior in the subsequent seven days.

### 2.2. The Behavior of Newborn Scorpions Climbing onto the Backs of Female Scorpions

To explore whether different female scorpions provided maternal care to fallen newborn scorpions, ten newborn scorpions per group were uniformly distributed in a moist-sand-covered plastic cup after the separation of the first-instar scorpions from their mothers, and we tracked how they climbed onto their mother’s back (MBS), a 7-day postpartum female scorpion’s back (PFS), and an antepartum scorpion’s back (AS). There were three experimental groups with five replicates per group, including a total of 150 newborn scorpions and 15 female scorpions. The time needed for each newborn scorpion to climb onto the back of the female adult scorpions was recorded from the first to the tenth scorpion.

### 2.3. The Molting Behavior of First-Instar Scorpions

Eighty litters of first-instar scorpions, for a total of 1691 scorpions, were divided into four groups for the molting behavior analyses: 390 newborn scorpions on their mother’s back (MB), 404 newborn scorpions with a 7-day postpartum scorpion (PF), 433 newborn scorpions with a dead and air-dried female scorpion (dead before one year) (DF), and 464 newborn scorpions on sand alone (SS). There were twenty mother scorpions, twenty 7-day postpartum scorpions, and twenty dead and air-dried female scorpions. Another ten litters of first-instar scorpions were divided into two equal parts, one of scorpions on their mother’s back (LMB) and the others on sand alone (LSS), to eliminate the possible effects of first-instar scorpions from different mothers on molting behavior. Subsequently, the molting behavior of all of the newborn scorpions was observed several times daily, and the survival rate of the first-instar scorpions was counted for seven days. The exuviae of the first-instar scorpions were collected, and their ultrastructure was examined by scanning electron microscopy.

### 2.4. The Aggregating Behavior of First-Instar Scorpions during the Molting Process

Different numbers of newborn scorpions, from one to ten newborns with five replicates per group, were prepared for observations of their aggregating behavior in the presence and absence of mother scorpions. There were a total of fifty mother scorpions in the experiment. The effect of newborn scorpion aggregating behavior on the survival rate was also tracked for seven days. To verify the correlation between the number and aggregate molting behavior of newborn scorpions, more newborn scorpions, ranging from one to five scorpions as an individual group with fifty replicates, were prepared for observations of their aggregating behavior on sand alone. Their aggregation states, which included one, two, three, four, and five aggregated newborn scorpions, were calculated. In addition, the exuviae of the first-instar scorpions were collected and analyzed by stereomicroscopy.

### 2.5. The Interplay between Newborn and Adult Male Scorpions

The differences in behavior between newborn and adult male scorpions were also analyzed. Twenty litters of first-instar scorpions were separated from their mothers and gently transferred to adult male scorpions. The ability of twenty litters of newborn scorpions (n = 372) to climb onto the backs of mother scorpions (MB) and to undergo aggregate molting was recorded over seven days. The behavior of adult male scorpions toward newborn scorpions was also recorded to evaluate the interplay among 385 newborn scorpions and twenty adult male scorpions (AM). The survival curves of newborn scorpions in the presence of mother scorpions and adult male scorpions for seven days were statistically drawn.

### 2.6. Calculation and Statistical Analysis

All experimental data were subjected to tests of the normal distribution and the homogeneity of variance by the Shapiro–Wilk test and Levene’s test in this work. Significant differences in the average time for newborn scorpions to climb onto the female scorpions’ backs were analyzed by using paired one-tailed *t*-tests. If the data disobeyed the normal distribution, statistical analyses were performed by using nonparametric tests to determine if there was any significant difference. The Kruskal–Wallis H test was used to determine if there was any significant difference in the survival rate of the first-instar scorpions in four distinct environments. The Wilcoxon signed-rank test was performed to determine if there was any significant difference between the survival rate of the first-instar scorpions from the same mother on their mother’s back and on sand alone, and from one to five scorpions on sand alone. SPSS 26.0 was used for significance testing, and *p* < 0.05 was regarded as statistically significant in a ≥95% confidence interval. Survival analyses were performed for the first-instar scorpions in the presence of mother scorpions and adult male scorpions with GraphPad Prism v8.0.

## 3. Results

### 3.1. Unique Feature of Aggregating Molting Behavior in First-Instar Scorpions

**Figure 1:**
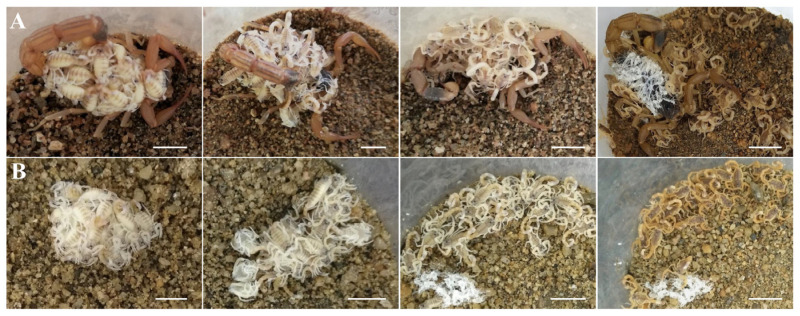
Aggregate molting behavior of first-instar scorpions. (**A**) Molting behavior of newborn scorpions aggregating on their mother’s back. (**B**) Aggregate molting behavior of newborn scorpions on sand. From left to right: newborn scorpions, molting scorpions, newly molted scorpions, and 3-day molted scorpions. Visible hardening and darkening were observed in first-instar scorpions after they shed their cuticle. Scale bar: 1.00 cm.

Unlike the independent molting behavior of second- and elder-instar scorpions, whole litters of first-instar *Mesobuthus martensii* scorpions preferred to attach to their mothers’ backs and aggregate at the time of molting (Figure 1A). Before ecdysis, it typically took approximately three days for them to grow and develop, and during this period, the newborn scorpions gradually adjusted their positions and postures to ensure that their heads were looking outside. The first-instar scorpions exhibited a molting pattern similar to the older scorpions: the cuticle ruptured at the side and front margins of the carapace, followed by the entire body, containing the chelicerae, pedipalps, and legs withdrawn from the exuviae. Due to the soft bodies, the pedipalps, legs or tails of the scorpions crosslinked with each other between adjacent scorpions during the molting process (see the second panel in Figure 1A). This crosslinking molting strategy allowed first-instar scorpion individuals to draw strength from their siblings for successful ecdysis due to remarkable aggregate molting behavior. This study is the first complete observation and description of the exceptional aggregate molting behavior of first-instar scorpions. The newly molted scorpions showed a strong preference for staying on their mother’s back (see the third panel in Figure 1A). Subsequently, a clump of exuviae, formed and crosslinked by the individual exuviae of the first-instar scorpions from aggregate molting, was observed on the back of the mother scorpion (see the fourth panel in Figure 1A).

**Figure 2:**
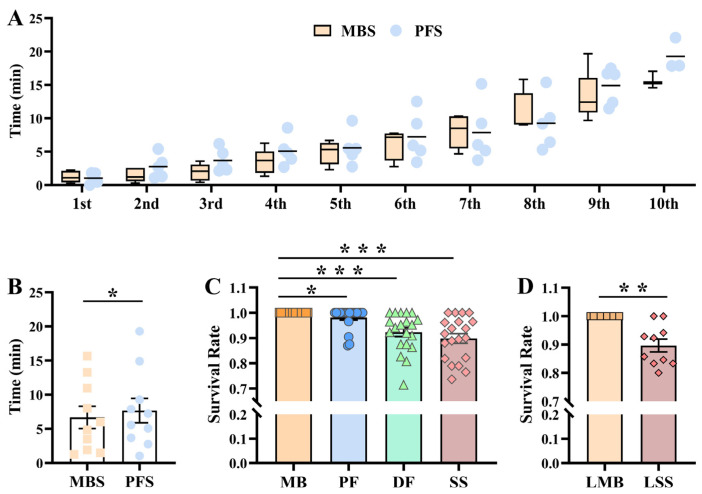
Special maternal care and the differential survival rates of first-instar scorpions in different environments during the molting process. (**A**) The time needed for each newborn scorpion to climb onto its mother’s back (MBS) or a 7-day postpartum female scorpion’s back (PFS) from the 1st to the 10th newborn scorpion. A group from the 1st to the 10th newborn scorpions with five replicates was selected for this study. (**B**) Average time for 10 newborn scorpions to climb onto their mothers’ backs (MBS) and 7-day postpartum female scorpions’ backs (PFS) under special maternal care. Paired one-tailed *t*-test. In every group of five replicates, n = 10. (**C**) Survival rate of 20 litters of newborn scorpions on their mothers’ backs (MB), 7-day postpartum female scorpions (PF), dead and air-dried female scorpions (DF), and sand (SS). Kruskal–Wallis H test. In every group of 20 replicates, n ≥ 12. (**D**) Survival rate of 10 litters of newborn scorpions, with each litter divided into two parts (one part on their mother’s back (LMB) and the other on sand alone (LSS)). Wilcoxon signed-rank test. In every group of 10 replicates, n ≥ 6. The error bars denote the standard errors. *: *p* < 0.05; **: *p* < 0.01; ***: *p* < 0.001.

The aggregating molting behavior was not completed for the first instars that lost the company of their mothers [20,21]. In the absence of mother scorpions, the first instars preferred to gather together rather than disperse on the sand (Figure 1B). During the molting process, the pedipalps, legs, or tails of the first-instar scorpions crosslinked with each other between adjacent scorpions (see the second panel in Figure 1B), which further indicated that an aggregative advantage was critical to molting. After these molted scorpions dispersed, a clump of exuviae from their aggregate molting remained (see the third and fourth panels in Figure 2B), which indicated that the molting ability of first-instar *Mesobuthus martensii* scorpions is independent of the mother.

### 3.2. Special Maternal Care Reduced the Time for First-Instar Scorpions to Climb onto Their Mother’s Back before Molting

In the presence and absence of mother scorpions, the whole first-instar litter successfully accomplished their molting via aggregate behavior (Figure 1), which prompted inquiry of the benefits that first-instar scorpions derived from their mothers in addition to the known function of protection against predators. To compensate for poor eyesight in scorpions, the trichobothria on the two pedipalps can probe moving objects around a scorpion [26]. With the assistance of the trichobothria, the mother scorpion was able to locate the mobile newborn scorpions on the sand and move to the newborn scorpions so that they could climb onto her back (Video S1). The amount of time each newborn scorpion spent climbing onto the back of the female scorpions was recorded for every group of ten newborn scorpions, with the mothers and 7-day postpartum female scorpions used for behavioral analysis (in every group of five replicates, n = 10). Interestingly, newborn scorpions were able to climb onto the mother’s back in different time intervals (Figure 2A). Unexpectedly, the 7-day postpartum female scorpions also provided maternal care to non-offspring newborn scorpions. In contrast to the obligatory task of the mother scorpions, the nonobligatory task of these 7-day postpartum scorpions generally took more time to complete for the newborn scorpions (*p* < 0.05; Figure 2B). The weaker maternal behavior of the 7-day postpartum female scorpion was reflected in detecting and helping the newborn scorpions climb to the back. Less climbing time was more helpful for prompting the aggregating behavior of newborn scorpions on their mothers’ backs to prepare for molting.

### 3.3. Maternal Care Increased the Survival Rate of First-Instar Scorpions during the Aggregate Molting Process

Most importantly, whether newborn scorpions successfully molt or not ultimately determines whether they will live or die. Therefore, four distinct environments, including newborn scorpions with the mother, newborn scorpions with a 7-day postpartum scorpion, newborn scorpions with a dead and air-dried adult female scorpion, and newborn scorpions with sand alone, were selected to examine the role of the mother scorpion in the survival of first-instar scorpions during the molting process. All of the newborn scorpions survived after the molting process when they were accompanied by mother scorpions, and an impressive survival rate of 98.07% ± 0.97% was even achieved in the presence of 7-day postpartum scorpions (in every group of 20 replicates, n ≥ 12, *p* < 0.05; Figure 2C). When the mother and 7-day postpartum scorpions were replaced with dead and air-dried female adult scorpions, the survival rate of the newborn scorpions decreased to 92.30% ± 1.72% (in every group of 20 replicates, n ≥ 12, *p* < 0.001; Figure 2C). This finding was further confirmed by the 89.83% ± 1.91% survival rate of newborn scorpions when newborn scorpions were all placed on sand alone (in every group of 20 replicates, n ≥ 12, *p* < 0.001; Figure 2C), which indicates the important role of the mother in the survival of newborn scorpions. These dead and air-dried female adult scorpions could not help newborn scorpions gather on her back, and three representative aggregating states of newborn scorpions were identified: all of the newborn scorpions on the back of the dead scorpion, some newborn scorpions on the back of the dead scorpion and other newborn scorpions on the sand, and all of the newborn scorpions on sand alone (Supplementary Figure S1). These various aggregating features suggested that the sand environment was harsher for the newborn scorpions than the backs of the adult female scorpions. To minimize the effect of different litters of newborn scorpions on their survival rate, ten litters of siblings were selected, and each litter was divided into two equal sets: one set was placed with the mother scorpions, and the other set was placed on the sand. Before and after the molting process, all of the newborn scorpions survived in the company of the mother scorpions, but only 89.63% ± 2.27% of the newborn scorpions survived without the company of the mother scorpions (in every group of 10 replicates, n ≥ 6, *p* < 0.01; Figure 2D). These results show that the litter of origin hardly influenced the survival rate of the newborn scorpions on the sand.

Overall, the survival rate of first-instar scorpions increased when a mother scorpion was present and was directly related to success in molting to the second instar. The role of the mother scorpion in the molting process of newborn scorpions could be largely replaced by 7-day postpartum scorpions but not by dead scorpions or sand alone.

### 3.4. The First-Instar Number Differentially Affected Aggregate Molting Behavior in the Presence/Absence of Maternal Care

During the molting process, all healthy newborn scorpions can survive under maternal care, which results in reduced time needed for first-instar scorpions to climb onto the mother’s back and provide a desirable molting environment. However, some newborn scorpions could not survive when placed with dead scorpions or on sand alone because they failed to assemble for aggregate molting (Figure 2 and Supplementary Figure S1). These differences indicate the significance of aggregating behavior in the successful molting of newborn scorpions.

**Figure 3:**
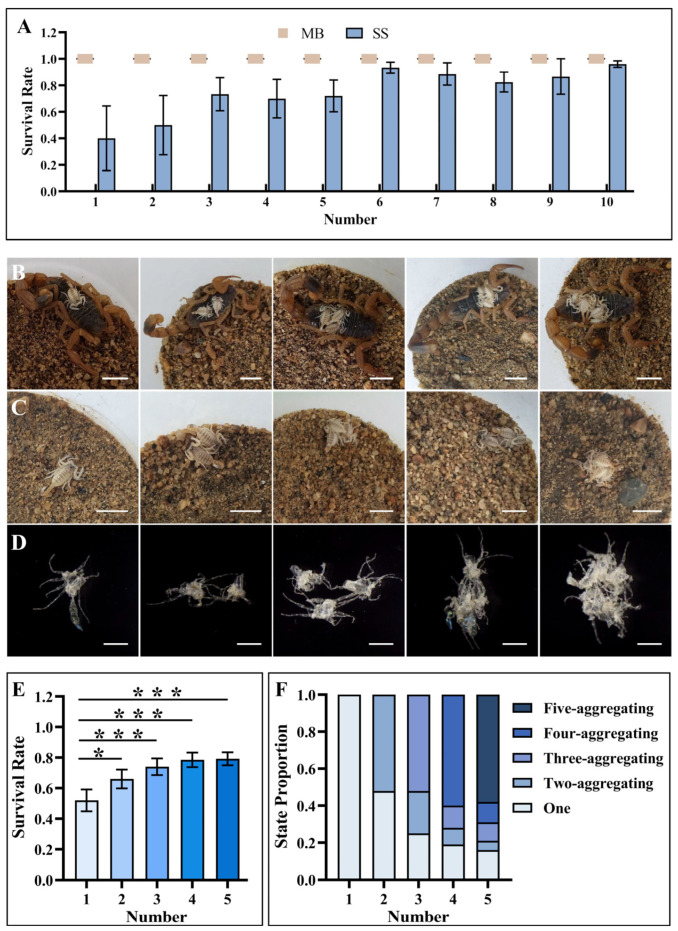
The effect of the quantity of first-instar scorpions on aggregate molting behavior and survival rate with/without maternal care. (**A**) Survival rate of first-instar scorpions, which were placed in groups ranging from 1 to 10 scorpions on the mother’s back (MB) or on sand alone (SS). In every group of five replicates, 1 ≤ n ≤ 10. (**B**,**C**) Representative aggregation of one to five newborn scorpions on their mother’s back and sand. (**D**) Microscope image of aggregates from one to five first-instar scorpion exuviae. (**E**) Survival rate of first-instar scorpions on sand alone in groups of one to five scorpions. The blue color of bar graph deepened with the number of the first-instar scorpions. Wilcoxon signed-rank test. In every group of 50 replicates, 1 ≤ n ≤ 5. (**F**) Different proportions of aggregating scorpion states consisting of one, two, three, four, and five aggregating states on sand alone during the molting process. The error bars denote the standard errors. Scale bars: 1.00 cm in B-C and 0.20 cm in D. *: *p* < 0.05; ***: *p* < 0.001.

As an important factor in the aggregating behavior, the different numbers of newborn scorpions were further investigated in the presence and absence of maternal care. Unexpectedly, a solitary newborn scorpion still survived after successful ecdysis on the mother’s back, even though it could not form a cluster for aggregate molting (Figure 3A). Successful ecdysis was achieved in two key steps. First, the newborn scorpion tried its best to stretch the pedipalps and legs, which seemed to grasp the back of the mother scorpion to obtain a stable hold to start molting. Second, the newly molted prosoma and mesosoma continued wiggling with the help of an unmolted metasoma connecting the exuviae anchored on the mother’s back before completing the molting process (Figure 3B and Supplementary Figure S2). In contrast to the 100% survival rate of a single newborn scorpion in the presence of the mother scorpion, only 40.00% ± 24.49% of newborn scorpions survived when a single newborn scorpion was placed on sand alone (in every group of five replicates, 1 ≤ n ≤ 10; Figure 3A), which further supported the critical role of the mother scorpion’s back in the molting process for newborn scorpions. As the number of newborn scorpions increased from one to ten, all of the scorpions formed a cluster on their mother’s back and survived after aggregate molting (Figure 3A). When the newborn scorpions were placed on sand alone without maternal care, the survival rate increased and fluctuated as the number of newborn scorpions increased (Figure 3A). To verify the correlation between the number of newborn scorpions and the aggregate molting behavior of newborn scorpions, survival experiments using newborn scorpions on sand alone and involving groups ranging from one to five scorpions were further performed with 50 replicates during the molting process. As shown in Figure 3E, the survival rate of the newborn scorpions stably increased from 52.00% ± 7.14% for a single newborn scorpion to 79.20% ± 4.24% for five newborn scorpions positioned on sand (two scorpions: *p* < 0.05; three scorpions: *p* < 0.001; four scorpions: *p* < 0.001; five scorpions: *p* < 0.001; in every group of 50 replicates, 1 ≤ n ≤ 5), which suggests the critical role of scorpion number in the aggregating behavior of newborn scorpions. An in-depth analysis revealed that the survival rates of newborn scorpions were closely related to their different aggregating states (Figure 3C,F). Among the five aggregating states, which included one, two, three, four, or five aggregating newborn scorpion groups, the proportion of one single scorpion as a group markedly decreased with the increase in the number of newborn scorpions, which decreased the mortality rate of newborn scorpions. In addition, approximately 58% of the scorpions could form a five-scorpion aggregating state, and approximately 26% of the scorpions could form two-, three-, or four-scorpion aggregating states when five newborn scorpions were positioned on sand alone (Figure 3F). These results further demonstrate that the tendency toward aggregate molting behavior significantly enhanced the chances of survival for newborn scorpions.

To elucidate why aggregate molting increased the survival rate of newborn scorpions, the structural features of their exuviae were characterized by using microscopy. Interconnection via the pedipalps and/or legs was observed among the exuviae from clusters of two, three, four, and five newborn scorpions. With the increase in the number of newborn scorpions, the interconnection degree became more complex due to the participation of the scorpion prosoma and mesosoma (Figure 3D). This exuvial crosslinking highlights the necessity of drawing a greater support advantage from the collaborative mechanism, which is essential for the molting behavior of newborn scorpions with their soft chitinous exoskeletons. The number of newborn scorpions greatly influenced the aggregate molting behavior, which helped to increase strength via collaborative arrangement. This strength was further enhanced by crosslinking between the newborn and mother scorpions so that even a single newborn scorpion could molt and survive on the mother’s back. Due to the limited aggregating ability of newborn scorpions in the absence of mother scorpions, the greater the number of newborn scorpions positioned on sand alone, the greater the survival rate of the newborn scorpions within a certain range.

### 3.5. Predatory Behavior of Adult Male Scorpions toward Newborn Scorpions

**Figure 4:**
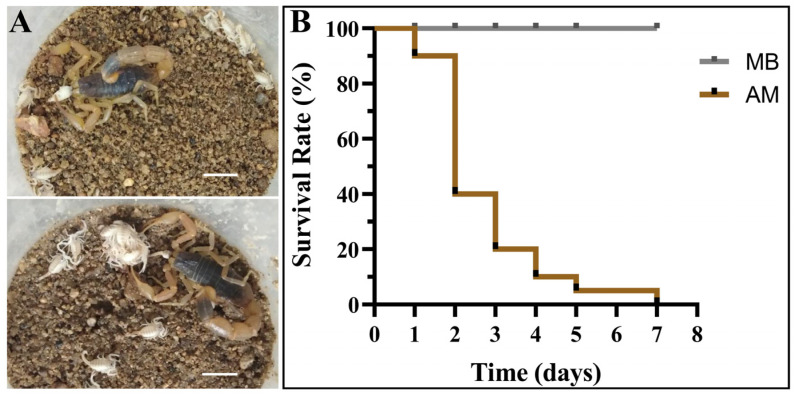
Survival status of newborn scorpions in the presence of adult male scorpions. (**A**) Representative predation of newborns by adult male scorpions. Scale bar: 1.00 cm. (**B**) Survival curve of newborn scorpions in presence of their mother’s back (MB) and adult male scorpions (AM). Every group of 20 replicates.

In addition to the maternal behavior of adult female scorpions, whether similar behavior occurred in adult male scorpions was also explored. Twenty litters of first-instar scorpions were separated from their mothers and transferred to the backs of adult male scorpions. When the newborn scorpions were placed with an adult male scorpion, they may not have been able to distinguish whether the male scorpion was or was not their mother and tried to climb onto its back. However, the male scorpion was not receptive to the behavior of the newborn scorpions and violently shook his body to make the newborn scorpions fall from its back. Moreover, adult male scorpions also preyed on these newborn scorpions (Figure 4A), which further affected the aggregating behavior of the newborn scorpions. When all the newborn scorpions successfully molted and survived from the twenty litters of first-instar scorpions in the presence of the mother scorpions, the survival curves of 385 newborn scorpions living with adult male scorpions revealed that the young scorpions rapidly died within the first three days (Figure 4B). The remaining living young scorpions also gradually died in the subsequent several days. These results suggest that adult male scorpions are not beneficial to the survival of newborn scorpions, which can be shown maternal behavior only by female adult scorpions.

## 4. Discussion

As an essential process, ecdysis must be experienced during the life history of arthropods. During the molting process, the new exoskeleton is too soft to provide protection against predators and conspecifics, and arthropods have developed different behaviors to overcome this problem. Independent molting behavior is widely adopted by most arthropods, and synchronous molting behavior is observed in modern social and sub-social groups of arthropods, such as spiders [4], shrimp [5], prawns [6], crabs, and springtails [7,8]. Synchronous molting behavior in arthropods is sometimes caused by stimuli from the physical environment, the food supply [4,5,27,28], and social facilitation [4,29].

**Figure 5:**
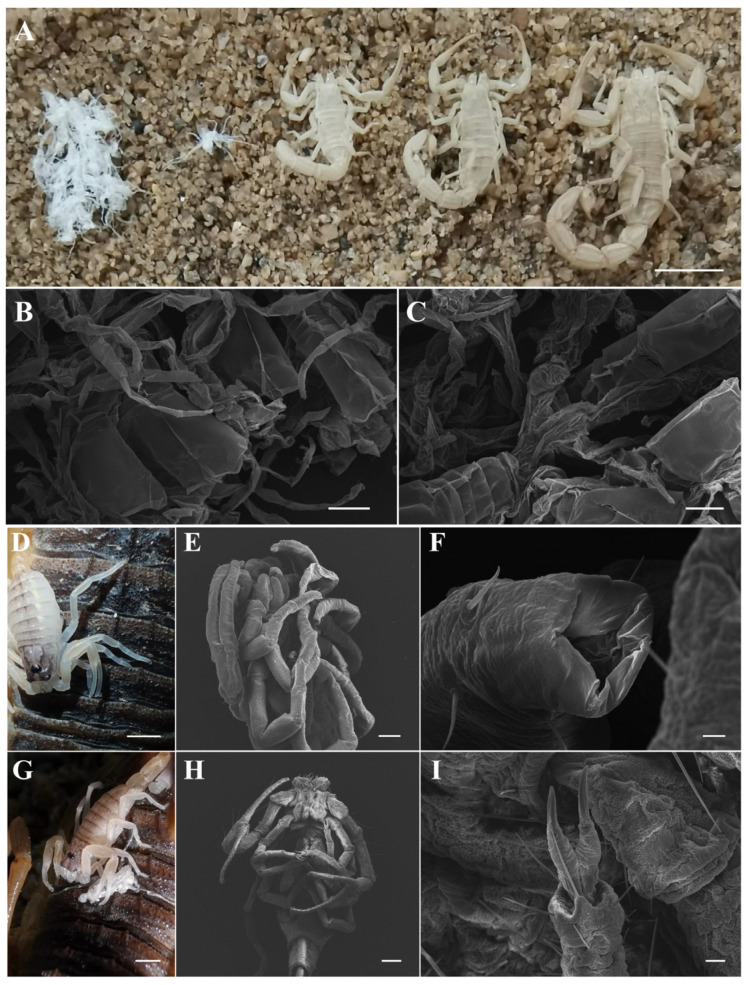
The exuvial morphology of scorpions and the differential structural features of leg ends from newborn scorpions before and after molting. (**A**) Exuvial morphology of scorpions of different instars from left to right: whole litter of first-instar scorpions, single first-instar scorpion, second-instar scorpion, third-instar scorpion, and fourth-instar scorpion. (**B**,**C**) Crosslinked morphology of different parts of first-instar exuviae under scanning electron microscope. (**D**) Morphological posture of pre-molting first-instar scorpion legs on their mother’s back. (**E**,**F**) Scanning electron micrographs of blunt cup-like tips of first-instar scorpion legs. (**G**) Morphology of legs of newly molted first-instar scorpions on their mother’s back. (**H**,**I**) Distal claws of newly molted first-instar scorpion legs determined by scanning electron microscopy. Scale bars: 1.00 cm in (**A**,**D**,**G**), 500 μm in (**B**,**C**,**E**,**F**), 50 μm in (**H**,**I**).

Scorpions are among the earliest terrestrial arthropods. Molting events have been described for extinct sea scorpions, which are the presumed ancestors of extant scorpions [17]. Through long-term evolution, the independent molting strategy has been adopted for second-instar and older scorpions, and the synchronous molting strategy is only used by newborn scorpions [20,21]. The individual exuviae of second and older instars retain a scorpion-like shape, whereas the exuviae from first-instar scorpions do not preserve a scorpion shape due to synchronous molting behavior (Figure 5A) [20,21,30]. In this work, the exuviae of a single newborn scorpion molting on its mother’s back were obtained, and the exuviae appeared octopus-shaped rather than scorpion-shaped (Figure 5A). Scanning electron microscopy revealed that the pedipalps, legs, or tails from the exuviae of first-instar scorpions crosslinked with each other between adjacent scorpions (Figure 5B,C), which highlights the importance of synchronous and collaborative molting strategies during the molting process of first-instar scorpions.

Maternal care is the first instance of social facilitation of the synchronous and collaborative molting behavior of first-instar scorpions. Traditionally, maternal care was regarded as an obligatory behavior for newborn scorpions [20,21,23,24]. In addition to the known protection from predators that maternal care provides, novel benefits of the synchronous and collaborative molting behavior were found in this work. First, the mother scorpions were always ready to help the fallen newborn scorpions climb onto their back again. The trichobothria of the mother scorpions were highly sensitive to active newborn scorpions on sand alone (Video S1). Since the trichobothria of newborn scorpions are rudimentary [31], the fallen newborn scorpions tended to walk around to find their mother and their siblings, which helped them form a cluster. Meanwhile, the mother scorpion and 7-day postpartum scorpions could use sensitive trichobothria to detect these fallen newborn scorpions and help them climb onto their back again (Figure 2B). Second, the back of the mother scorpion is the evolutionarily preferred location for newborn scorpion molting. The newborn scorpions always successfully molted regardless of the number of newborn scorpions in the aggregated group (Figure 3). More importantly, a 100% survival rate could be achieved for a single newborn scorpion on the mother’s back after successful molting, which significantly decreased to 52.00% ± 7.14% for a single newborn scorpion on sand alone due to unsuccessful molting (in every group of 50 replicates, 1 ≤ n ≤ 5; Figure 3E,F). Their molting behavior indicates that a single newborn scorpion could stretch its pedipalps and legs and clutch the back of the mother scorpion (Supplementary Figure S2). These marked differences demonstrate that the mother scorpion’s back is an ideal habitat for newborn scorpion molting. The biological roles of the mother scorpions were rarely observed in 15-day postpartum scorpions. The newborn scorpions were not allowed to stay on the back of the 15-day postpartum scorpions, and some newborn scorpions were preyed upon by the 15-day postpartum scorpions (Supplementary Figure S3). These findings reveal that the period of maternal behavior, both in helping the fallen newborns to climb onto their back and in providing their back as a desirable molting environment, was limited to the postpartum scorpions. Such maternal behavior is distinct from that of the adult female spider *Amaurobius ferox,* which is willing to eat young spiders during the second ecdysis [4]. However, whether this novel maternal behavior in *Mesobuthus martensii* scorpions occurs in other scorpion species remains to be investigated.

The collaborative benefit is another innovative feature of the synchronous and aggregate molting behavior of newborn scorpions. The cuticle of newborn scorpions significantly differed from the hardened cuticles that contribute to the individual molting behavior of older scorpions (Figure 5). When a single newborn scorpion started to molt, it stretched its pedipalps and legs and tightly grasped the mother scorpion’s back (Supplementary Figure S2), which caused an indistinguishable scorpion-shaped exuviae to detach from a single newborn scorpion and a clump of exuviae from many newborn scorpions (Figure 5A). The microscopy analysis clearly revealed that the pedipalps, legs, or tails of the first-instar exuviae from one, two, three, four, and five newborn scorpions crosslinked well with each other between adjacent scorpions (Figure 5B,C). When more newborn scorpions gathered and molted together, a more complex interconnection occurred among the exuviae (Figure 5A). These unique characteristics strongly indicate that an interconnection advantage is essential for the molting behavior of newborn scorpions. When it was difficult for a newborn scorpion on sand alone to obtain such extra support strength during the molting process, approximately half of the scorpions were unable to survive (Figure 3A). With the help of extra support from the crosslinked exuviae among more newborn scorpions on sand alone, their survival rate gradually improved (Figure 3A,E). These findings clearly support that newborn scorpions with their soft cuticles help each other during molting activities. This social facilitation of synchronous molting behavior is also different from the cooperative prey capture by the young spider *Amaurobius ferox* during the early molting stage [4].

The unique molting behavior of newborn scorpions could maximize the benefits of maternal care and reciprocal aggregation. This synchronous molting behavior depends on the cup-like tips of first-instar scorpion legs. Usually, the distal claws of the eight legs are the classic structural feature of scorpions [20,21]. Unlike the classic distal claws of scorpion legs, the distal ends of newborn scorpion legs maintain a calathiform state (Figure 5F) [31]. The newborn scorpion legs function like a suction cup to support their body hanging on the edge of the mother scorpion’s back (Supplementary Figure S2 and Video S2). Using their calathiform legs, some newborn scorpions were able to tightly grasp the mother scorpion, and some newborn scorpions could tightly grasp adjacent newborn scorpions due to their very soft exoskeletons. Once the first scorpions completed molting, the classic distal claws of the scorpion legs appeared (Figure 5G–I) [31], which may result in the molting behavior switching from aggregate molting to individual molting in older scorpions (Figure 5A). The elder scorpions with hardened exoskeletons could support themselves by their legs with the hardened exoskeletons and did not need the distal claws during the molting process.

## 5. Conclusions

Newborn scorpions exhibit special aggregate molting behavior, which increases their chances of survival. This study is the first complete observation and experimental study on the aggregate molting behavior of first-instar scorpions. The results of this study expand our knowledge of animal molting behavior. These aggregating groups of newborn scorpions are considerably advantageous because they save mother scorpions the time spent searching for newborn scorpions and satisfy the need of newborn scorpions to climb onto their mother’s back. Even when these groups of newborn scorpions lose the chance to molt on their mother’s back, most still complete their molting activities and survive. During the molting process, the aggregating behavior of the newborn scorpions greatly contributes to collaborative molting via the crosslinking of pedipalps, legs, or tails among the scorpions, whether they are on the mother scorpion’s back or not. Unusual aggregating behavior could indicate an evolutionary role over hundreds of millions of years in scorpions. The combined advantages of maternal care and the cooperative nature of aggregate molting behavior in first-instar scorpions play an essential role in survival and reveal the unusual survival strategy of arthropods during molting.

## Data Availability

Data are contained within the article or Supplementary Materials.

## Supplementary Materials

The following are available online at https://www.mdpi.com/article/10.3390/insects15090726/s1, Figure S1: The aggregate molting behavior of first-instar scorpions with dead and air-dried female scorpions, Figure S2: The molting behavior of a single newborn scorpion on its mother’s back, Figure S3: Representative aggregate molting behavior of first-instar scorpions with 15-day postpartum scorpions, Video S1: Special maternal care in helping first-instar scorpions climb onto their mother’s back, Video S2: The molting behavior of a single newborn scorpion on its mother’s back.
