# Supplementary material for: Special Survival Strategy of First-Instar Scorpions Revealed by Synchronous Molting Behavior from Social Facilitation of Maternal Care and Reciprocal Aggregation
Source: Insects. 2024 Sep 21;15(9):726. doi: 10.3390/insects15090726 (PMC11432028; doi:10.3390/insects15090726)
Supplement: Supplementary file 1 [file insects-15-00726-s001.zip › Supplementary Materials.pdf]

Supplementary Materials

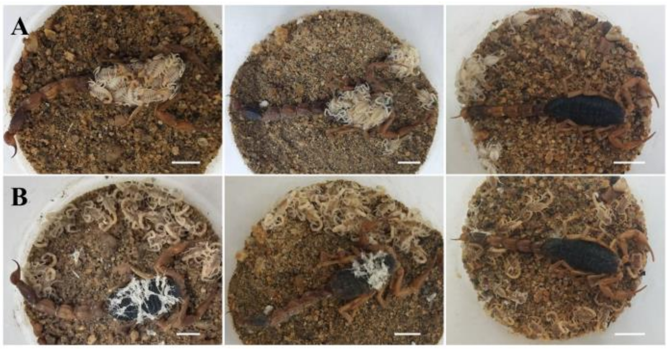

Supplementary Figure S1. The aggregate molting behavior of the first-instar scorpions with the dead and air-dried female scorpions

(A) The aggregate molting behavior of pre-molting newborn scorpions in the presence of dead and air-dried female scorpions. From left to right: all the newborn scorpions on the back; some on the back, others on the sand; and all on the sand before molting. (B) All 3-day molted scorpions on sand alone living with the dead and air-dried female scorpion. From left to right: almost all the exuviae on the adult scorpion back, partial exuviae on the adult scorpion back, and all the exuviae on the sand. Scale bar: 1.00 cm in A and B.

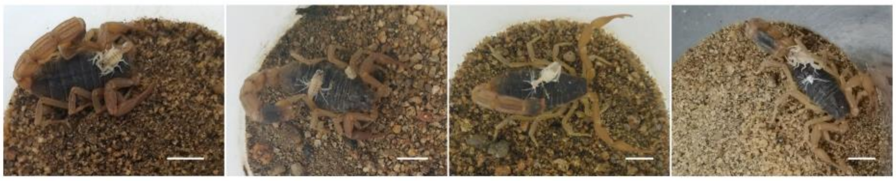

Supplementary Figure S2. The molting behavior process of a single newborn scorpion on the mother's back

The molting process of the newborn scorpion from left to right: on the mother's back before molting, the crevice of the head with pedipalps and legs adhering to the mother's back during molting, the wiggling of the prosoma and mesosoma with the metasoma

connecting the exuvia, the second-instar scorpion and its exuvia on the mother's back after molting. Scale bar: 1.00 cm.

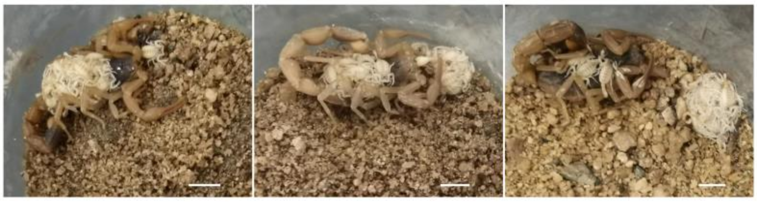

Supplementary Figure S3. Representative aggregate molting behavior of the first-instar scorpions with the 15-day postpartum scorpion

Representative aggregating states of the first-instar scorpions with the 15-day postpartum scorpions during the molting process. From left to right: almost all the newborn scorpions on the 15-day postpartum scorpion back, nearly half of the newborn scorpion on the sand, while the 15-day postpartum scorpion prey on a newborn scorpion, almost all the newborn scorpions on the sand. Scale bar: 1.00 cm.

Video S1. Special maternal care in helping the first-instar scorpions to climb onto the mother's back

Video S2. The molting behavior of a single newborn scorpion on the mother's back
